# Outcomes of Acute Chest Syndrome in Adult Patients with Sickle Cell Disease: Predictors of Mortality

**DOI:** 10.1371/journal.pone.0094387

**Published:** 2014-04-16

**Authors:** Veerajalandhar Allareddy, Aparna Roy, Min Kyeong Lee, Romesh P. Nalliah, Sankeerth Rampa, Veerasathpurush Allareddy, Alexandre T. Rotta

**Affiliations:** 1 Assistant Professor of Pediatrics, Pediatric Critical Care, Rainbow Babies and Children's Hospital, Cleveland, Ohio, United States of America; 2 Fellow, Pediatric Critical Care Medicine, Rainbow Babies and Children's Hospital, Cleveland, Ohio, United States of America; 3 Developmental Biology, Harvard School of Dental Medicine, Boston, Massachusetts, United States of America; 4 Instructor, Dental Medicine, Harvard University, Boston, Massachusetts, United States of America; 5 Advanced Graduate Student, Texas A & M University, College station, Texas, United States of America; 6 Associate Professor, Department of Orthodontics, University of Iowa; Iowa, United States of America; 7 Professor of Pediatrics, Pediatric Critical Care, Rainbow Babies and Children's Hospital, Cleveland, Ohio, United States of America; S.G.Battista Hospital, Italy

## Abstract

Adults with sickle cell disease(SCD) are a growing population. Recent national estimates of outcomes in acute chest syndrome(ACS) among adults with SCD are lacking. We describe the incidence, outcomes and predictors of mortality in ACS in adults. We hypothesize that any need for mechanical ventilation is an independent predictor of mortality.

**Methods:**

We performed a retrospective analysis of the Nationwide Inpatient Sample(2004–2010),the largest all payer inpatient database in United States, to estimate the incidence and outcomes of ACS needing mechanical ventilation(MV) and exchange transfusion(ET) in patients >21 years. The effects of MV and ET on outcomes including length of stay(LOS) and in-hospital mortality(IHM) were examined using multivariable linear and logistic regression models respectively. The effects of age, sex, race, type of sickle cell crisis, race, co-morbid burden, insurance status, type of admission, and hospital characteristics were adjusted in the regression models.

**Results:**

Of the 24,699 hospitalizations, 4.6% needed MV(2.7% for <96 hours, 1.9% for ≥96 hours), 6% had ET, with a mean length of stay(LOS) of 7.8 days and an in-hospital mortality rate(IHM) of 1.6%. There was a gradual yearly increase in ACS hospitalizations that needed MV(2.6% in 2004 to 5.8% in 2010). Hb-SS disease was the phenotype in 84.3% of all hospitalizations. After adjusting for a multitude of patient and hospital related factors, patients who had MV for <96 hours(OR = 67.53,p<0.01) or those who had MV for ≥96 hours(OR = 8.73,p<0.01) were associated with a significantly higher odds for IHM when compared to their counterparts. Patients who had MV for ≥96 hours and those who had ET had a significantly longer LOS in-hospitals(p<0.001).

**Conclusion:**

In this large cohort of hospitalized adults with SCD patients with ACS, the need for mechanical ventilation predicted higher mortality rates and increased hospital resource utilization. Identification of risk factors may enable optimization of outcomes.

## Introduction

Survival of children with sickle cell disease well into adulthood is commonplace in the current era, and due, at least in part, to advances in comprehensive care of these patients [1]. In fact, a landmark study performed nearly 20 years ago indicated that approximately 50 percent of patients with sickle cell disease survived beyond the fifth decade of life [2]. Acute chest syndrome is a major complication of sickle cell disease and a significant cause of morbidity and mortality in these patients [2], [3], [4]. Approximately 50 percent of patients with sickle cell disease will have an episode of acute chest syndrome during their lifetime [5], and mortality related to these episodes is four times higher in adults compared to children [5], [6].

A recent study of the effect of hospital type and provider specialty on outcomes of hospitalized adolescents and young adults (16–25 years) with sickle cell disease and acute chest syndrome found that general hospitals carry higher intubation risks for adolescents and young adults with sickle cell disease and acute chest syndrome compared with children's hospitals [7]. The impact of the need for mechanical ventilation on outcomes in adult patients with sickle cell disease and respiratory failure due to acute chest syndrome is unknown.

Although, there are no randomized trials demonstrating the optimal treatment of acute chest syndrome in adults with sickle cell disease, transfusion therapy- especially exchange transfusion- has remained the cornerstone of management in moderate to severe cases of acute chest syndrome in several centers [8], [9]. National estimates of exchange transfusion usage and its impact on outcomes, such as in-hospital mortality and length of stay, are largely unknown. Patient and hospital level predictors of mortality in acute chest syndrome in sickle cell disease adult patients are also lacking. As such, identification of risk factors can help assess prognosis and devise preventive strategies to optimize outcomes.

The objective of our study is to estimate the incidence and outcomes of acute chest syndrome needing mechanical ventilation and exchange transfusion in adult patients (>21 years of age) with sickle cell crisis. We hypothesize that the need for mechanical ventilation predicts higher mortality in these patients.

## Materials and Methods

### Design and description of the database

We performed a retrospective analysis of the Nationwide Inpatient Sample (NIS) for the years 2004 to 2010. The NIS is the largest ***inpatient*** all-payer hospital discharge database in the United States that is a part of the Healthcare Cost and Utilization Project (HCUP) sponsored by the Agency for Healthcare Research and Quality (AHRQ) [10]. The Nationwide Inpatient Sample contains data of all nonfederal acute-care general hospitals in the United States and has information on a multitude of patient and hospital level variables including age, gender, race, reason for hospitalization, secondary diagnoses, procedures performed during hospitalization, length of stay in-hospital, hospital charges, type of admission, insurance status, and hospital level variables (teaching status, bed size, and geographic location).

### Data user agreement

The first author (VJA) completed the data user agreement with HCUP-AHRQ and obtained the patient/hospital ***de-identified*** data. As per University Hospitals Case Medical Center institutional review board (IRB) and in agreement with Federal Regulations 45 CFR 46.101 (b) which states “*research involving the collection or study of existing data, documents, records, pathological specimens, or diagnostic specimens, if these sources are publicly available or if the information is recorded by the investigator in such a manner that subjects cannot be identified, directly or through identifiers linked to the subjects*,” such studies are permitted to be classified as research that is “exempt” from IRB full or expedited review. IRB was not consulted for approval since the current study was a retrospective analysis of hospital based discharge dataset that is available publicly for purchase from Agency for Healthcare Research and Quality (AHRQ). The HCUP-AHRQ data user agreement precludes us from reporting individual cell counts ≤10 to preserve patient confidentiality. Consequently, these numbers were not reported in our study.

### Selection of cases and outcome variables examined

All adult hospitalizations (age >21 years) with a diagnosis of acute chest syndrome (International Classification of Disease, 9th edition, clinical modification [ICD-9-CM] codes of 517.3) with sickle cell crisis (ICD-9-CM codes of 282.42, 282.62, 282.64, and 282.69) were selected. The NIS dataset has 25 diagnostic fields and we used all these fields to select cases. For several cases, acute chest syndrome is not the primary reason for hospitalization, but develops during the hospitalization. Consequently, both elective and non-elective admissions were selected for analysis. Performance of continuous invasive mechanical ventilation (MV [ICD-9-CM procedure codes of 96.70, 96.71, and 96.72]) and exchange transfusion (ET [procedure code of 99.01]) was obtained for these hospitalizations. Co-morbid burden was estimated by using the NIS-disease severity files. The number of co-morbid conditions was computed for each hospitalization. The types of co-morbid conditions examined included: AIDS, alcohol abuse, deficiency anemias, rhematoid arthritis/collage vascular diseases, chronic blood loss anemia, congestive heart failure, chronic pulmonary disease, coagulopathy, depression, diabetes – uncomplicated, diabetes with chronic complications, drug abuse, hypertension, hypothyroidism, liver disease, lymphoma, fluid and electrolyte disorders, metastatic cancer, neurological disorders, obesity, paralysis, peripheral vascular disorders, psychoses, pulmonary circulation disorders, renal failure, solid tumor without metastasis, peptic ulcer disease, valvular disease, and weight loss. Seasonal variations in outcomes were described using simple descriptive statistics.

Acute chest syndrome hospitalizations needing mechanical ventilation, exchange transfusion and in-hospital mortality were examined yearly for trends (2004 to 2010). The independent variables of interest included a set of factors at both patient level (age, gender, type of admission, insurance status, race/ethnicity, type of sickle cell crisis, and presence of co-morbid conditions) and hospital level (hospital teaching status, bed size, and hospital location). The type of sickle cell crisis examined included Sickle-cell thalassemia with crisis (ICD 9 CM Code 282.42), Hb-SS disease with crisis (282.62), Sickle-cell/HB-C disease with crisis (282.64), and other sickle-cell disease with crisis (282.69).

### Analytical approach

The effects of mechanical ventilation and exchange transfusion on outcomes including length of stay (LOS) and in-hospital mortality were examined using multivariable linear and logistic regression models respectively. Since length of stay was skewed, it was log transformed and used as the dependent variable in the regression model. Taylor Linearization Methods was used to estimate the standard errors. In the multivariable logistic regression model for predicting in-hospital mortality, the odds ratios and 95% confidence intervals were computed for each level of independent variable. In both multivariable regression models, the effects of clustering of outcomes within-hospitals were accounted for. The effects of age, sex, type of sickle cell crisis, race, co-morbid burden, insurance status, type of admission, and hospital characteristics were adjusted in the regression models. All statistical analyses were conducted using SAS Version 9.3 (SAS Institute, Cary, NC) and SUDAAN Version 10.0.1 (Research Triangle Park, NC). All statistical tests were two-sided and a p-value of <0.05 was deemed to be statistically significant.

## Results

**Figure 1:**
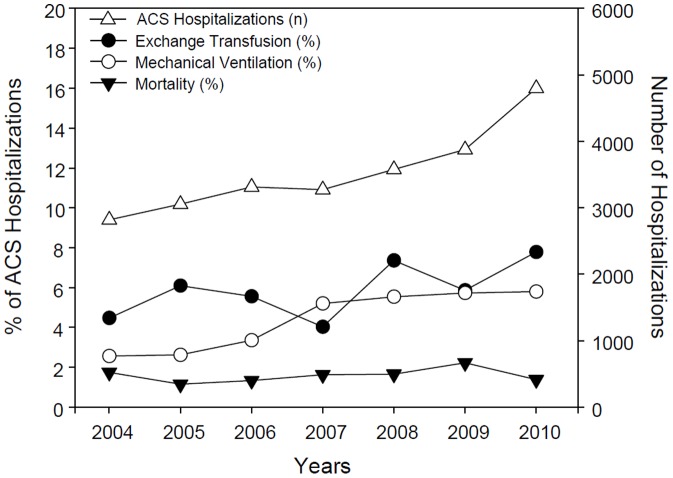
Acute Chest Syndrome Hospitalizations and Outcomes per year. The descriptive figure shows the number of acute chest syndrome hospitalizations per year- identified by “n” (2004 to 2010)- Right y axis. Outcomes such as Exchange transfusion(%), Mechanical Ventilation(%) and Mortality(%) are shown as percentages of acute chest syndrome hospitalizations.(2004 to 2010)- Left y axis.

There were a total a total of 24,699 hospitalizations for sickle cell crisis with acute chest syndrome (range is 2,818 hospitalizations in year 2004 to 4,796 hospitalizations in year 2010) See Figure 1. Hb-SS disease was the phenotype in 84.3% of all hospitalizations, while 4.2% had sickle-cell thalassemia, 2.5% had sickle-cell/Hb-C disease, and 9.8% had other forms of sickle-cell disease (Table 1). Close to 2.7% of patients required mechanical ventilation for less than 96 consecutive hours, 1.9% had mechanical ventilation for 96 consecutive hours or more, and 6% had an exchange transfusion. There was a gradual yearly increase in the number of acute chest syndrome hospitalizations that needed mechanical ventilation. Use of exchange transfusions varied on yearly basis with the lowest being in 2007, the year when mechanical ventilation use was higher than exchange transfusions - See figure 1.

**Table 1:** Type of sickle cell crisis and procedures performed.

**Type of sickle cell crisis (ICD-9-CM Code)**	**N (%)**
Sickle-cell thalassemia with crisis (282.42)	1029 (4.2%)
Hb-SS disease with crisis (282.62)	20814 (84.3%)
Sickle-cell/HB-C disease with crisis (282.64)	629 (2.5%)
Other sickle-cell disease with crisis (282.69)	2415 (9.8%)
**Procedures (ICD-9-CM procedure code)**	
Continuous invasive mechanical ventilation for less than 96 consecutive hours (96.71)	665 (2.7%)
Continuous invasive mechanical ventilation for 96 consecutive hours or more (96.72)	468 (1.9%)
Exchange transfusion (99.01)	1489 (6%)

(N = 24699).

The characteristics of hospitalizations are summarized in table 2. The mean patient age was 33 years and males comprised 48.6% of all hospitalizations. A total of 20,089 hospitalizations had information on race. Of these, 92.3% were classified as blacks, 4.3% as Hispanics, 1.4% as whites, 0.3% as Asians/pacific islanders, 0.2% as Native Americans, and 1.4% were other races. A concomitant co-morbid condition was present in 63.7% of all hospitalizations. Close to 95.5% of all hospitalizations occurred on an emergent or urgent basis. About 29.6% of the hospitalizations were covered by Medicare, 40.5% by Medicaid, 20.2% by private insurance, and 3% by other insurance plans. About 6.7% were uninsured. About 73.5% were treated in teaching hospitals. Large bed size hospitals treated 72% of hospitalizations.

**Table 2:** Characteristics of hospitalizations.

Characteristic	Response	N (%)
Sex	Male	11999 (48.6%)
	Female	12696 (51.4%)
Race	White	288 (1.4%)
	Black	18552 (92.3%)
	Hispanic	871 (4.3%)
	Asian/Pacific Islander	55 (0.3%)
	Native American	44 (0.2%)
	Other/Mixed races	278 (1.4%)
Insurance	Medicare	7300 (29.6%)
	Medicaid	9976 (40.5%)
	Private	4978 (20.2%)
	Uninsured	1643 (6.7%)
	Other insurance	738 (3%)
Type of admission	Emergency/Urgent	23556 (95.5%)
	Elective	1099 (4.5%)
Number of co-morbid conditions	0	8967 (36.3%)
	1	7694 (31.1%)
	2	4414 (17.9%)
	3	2032 (8.2%)
	4	974 (3.9%)
	5	414 (1.7%)
	6	155 (0.6%)
	7	35 (0.1%)
	> = 8	13 (0.05%)
Types of co-morbid conditions	AIDS	89 (0.4%)
	Alcohol abuse	241 (1%)
	Deficiency anemias	556 (2.2%)
	Rhematoid arthritis/collage vascular diseases	278 (1.1%)
	Chronic blood loss anemia	144 (0.6%)
	Congestive heart failure	1699 (6.9%)
	Chronic pulmonary disease	3546 (14.3%)
	Coagulopathy	1294 (5.2%)
	Depression	1596 (6.5%)
	Diabetes – uncomplicated	648 (2.6%)
	Diabetes with chronic complications	69 (0.3%)
	Drug abuse	1742 (7%)
	Hypertension	3552 (14.4%)
	Hypothyroidism	280 (1.1%)
	Liver disease	577 (2.3%)
	Lymphoma	54 (0.2%)
	Fluid and electrolyte disorders	6221 (25.2%)
	Metastatic cancer	0
	Neurological disorders	1102 (4.5%)
	Obesity	559 (2.3%)
	Paralysis	294 (1.2%)
	Peripheral vascular disorders	106 (0.4%)
	Psychoses	595 (2.4%)
	Pulmonary circulation disorders	2564 (10.4%)
	Renal failure	1023 (4.1%)
	Solid tumor without metastasis	25 (0.1%)
	Peptic ulcer disease	DS
	Valvular disease	721 (2.9%)
	Weight loss	292 (1.2%)
Hospital teaching status	Non-teaching status	6472 (26.5%)
	Teaching status	17963 (73.5%)
Hospital bed size	Small	1711 (7%)
	Medium	5128 (21%)
	Large	17596 (72%)
Hospital region	Northeast	6492 (26.3%)
	Midwest	4289 (17.4%)
	South	11530 (46.7%)
	West	2388 (9.7%)
Age in years	Mean	33.2
	Standard error	0.19

DS: Discharge information is suppressed as individual cell count is < = 10 (as per data user agreement with HCUP-AHRQ).

Disposition status following hospitalization is summarized in table 3. Routine discharge was the outcome in 86.2% of the hospitalizations, while, 1.7% were transferred to another acute care hospital, 1.9% were transferred to a long term care facility, 5.3% were discharged to a home health care facility, and 3.2% were discharged against medical advice. A total of 393 patients died in-hospitals (mortality rate of 1.6%).

**Table 3:** Disposition status.

Disposition status	N (%)
Routine discharge	21287 (86.2%)
Transfer to another acute care facility	431 (1.7%)
Transfer to another long term care facility (eg. Skilled nursing facility)	464 (1.9%)
Home health care	1313 (5.3%)
Discharged against medical advice	793 (3.2%)
In-hospital mortality	393 (1.6%)

Results of the multivariable analysis examining predictors of in-hospital mortality are summarized in table 4. Patients who had mechanical ventilation for less than 96 consecutive hours (OR = 67.53, 95% CI = 34.12–133.63, p<0.01) or those who had mechanical ventilation for 96 consecutive hours or more (OR = 8.73, 95% CI = 2.16–35.24, p<0.01) were associated with a significantly higher odds for in-hospital mortality when compared to those who did not require mechanical ventilation.

**Table 4:** Predictors of In-hospital mortality.

Characteristic	Response	OR (95% CI)	p-value
Type of sickle cell crisis	Hb-SS disease with crisis	0.71 (0.33–1.51)	0.37
	All other crisis	Reference	
Procedure	Continuous invasive mechanical ventilation for <96 consecutive hours	67.53 (34.12–133.63)	<0.001
	Continuous invasive mechanical ventilation for ≥96 consecutive hours	8.73 (2.16–35.24)	<0.001
	Exchange transfusion	0.13 (0.02–1.16)	0.07
Age in years	Each 1 year increase in age	1.02 (0.99–1.06)	0.17
Sex	Female	0.67 (0.33–1.35)	0.26
	Male	Reference	
Race	Black	0.27 (0.05–1.49)	0.13
	White	Reference	
Type of admission	Elective	0.86 (0.15–4.95)	0.87
	Emergency/Urgent	Reference	
Comorbid conditions	Each 1 unit increase in co-morbid conditions	1.17 (0.97–1.41)	0.11
Insurance status	Medicare	1.21 (0.24–6.06)	0.82
	Medicaid	2.08 (0.41–10.46)	0.38
	Private insurance	2.04 (0.43–9.77)	0.37
	Other insurance	0.70 (0.03–14.94)	0.82
	Uninsured	Reference	
Teaching status of hospital	Teaching	1.46 (0.67–3.21)	0.34
	Non teaching	Reference	
Hospital bed size	Large bed	0.95 (0.48–1.91)	0.89
	Small/Medium	Reference	
Hospital region	Northeast	0.78 (0.23–2.65)	0.69
	Midwest	1.09 (0.28–4.30)	0.90
	South	0.58 (0.18–1.85)	0.36
	West	Reference	

The mean hospital LOS was 7.8 days. Results of the multivariable analysis examining predictors of length of stay are summarized in table 5. Patients who had mechanical ventilation for 96 consecutive hours or more (estimate  = 0.9252, 95% CI = 0.81816–1.0318, p<0.001) and those who had an exchange transfusion had a significantly longer hospitalizations (estimate  = 0.4033 95% CI = 0.3120–0.4947, p<0.001) after adjusting for all other patient and hospital related factors. Increase in age was associated with shorter hospital length of stay (estimate  = −0.0066, 95% CI = −0.0094–−0.0038, p<0.001). Females were associates with longer length of stay compared to males (estimate  = 0.0880, 95% CI = 0.0376–0.1384, p<0.001). Patients hospitalized on an elective basis had longer length of stay (estimate  = 0.2428 95% CI = 0.1364–0.3492, p<0.001) compared to those hospitalized on an emergent or urgent basis. Those covered by Medicaid (estimate  = 0.0940, 95% CI = 0.0009–0.1871, p = 0.048) and other insurance plans (estimate  = 0.1974, 95% CI = 0.0561–0.3386, p = 0.006) were associated with longer length of stay compared to the uninsured. Large bed size hospitals were associated with longer length of stay compared to small/medium bed size hospitals (estimate  = 0.0655 95% CI = 0.0033–0.1277, p = 0.04). Seasonal variation in acute chest syndrome hospitalizations, mechanical ventilation and in-hospital mortality is shown in table 6.

**Table 5:** Predictors of length of stay in-hospital.

Characteristic	Response	Estimate* (95% CI)	p-value
Type of sickle cell crisis	Hb-SS disease with crisis	−0.0054 (−0.0769–0.0660)	0.88
	All other crisis	Reference	
Procedure	Continuous invasive mechanical ventilation for <96 consecutive hours	0.0398 (−0.1290–0.2087)	0.64
	Continuous invasive mechanical ventilation for ≥96 consecutive hours	0.9252 (0.81816–1.0318)	<0.001
	Exchange transfusion	0.4033 (0.3120–0.4947)	<0.001
Age in years	Each 1 year increase in age	−0.0066 (−0.0094–−0.0038)	<0.001
Sex	Female	0.0880 (0.0376–0.1384)	<0.001
	Male	Reference	
Race	Black	−0.0373 (−0.2424–0.1678)	0.72
	Hispanic	−0.1428 (−0.3721–0.0866)	0.22
	Asian/Pacific islanders	−0.1910 (−0.5377–0.1557)	0.28
	Native Americans	−0.0619 (−0.4368–0.3129)	0.75
	Other races	0.1458 (−0.1217–0.4132)	0.28
	White	Reference	
Type of admission	Elective	0.2428 (0.1364–0.3492)	<0.001
	Emergency/Urgent	Reference	
Co-morbid conditions	Each 1 unit increase in co-morbid conditions	0.1062 (0.0864–0.1260)	<0.001
Insurance status	Medicare	0.0870 (−0.0145–0.1886)	0.09
	Medicaid	0.0940 (0.0009–0.1871)	0.048
	Private insurance	0.0109 (−0.0947–0.1164)	0.84
	Other insurance	0.1974 (0.0561–0.3386)	0.006
	Uninsured	Reference	
Teaching status of hospital	Teaching	0.0560 (−0.0106–0.1226)	0.10
	Non teaching	Reference	
Hospital bed size	Large bed	0.0655 (0.0033–0.1277)	0.04
	Small/Medium	Reference	
Hospital region	Northeast	0.0348 (−0.0709–0.1406)	0.52
	Midwest	0.0219 (−0.0945–0.1384)	0.71
	South	0.0582 (−0.0428–0.1591)	0.26
	West	Reference	

Mean length of stay in-hospital is 7.8 days (standard error is 0.13)

*Positive estimate implies increased LOS compared to the reference variable. Negative estimate implies decreased LOS compared to the reference variable.

**Table 6:** Sensitivity Analysis to Examine Seasonal Variations.

Variable	January–March	April–June	July–September	October–December
Sickle-cell thalassemia with crisis (282.42)	291	172	265	301
Hb-SS disease with crisis (282.62)	5336	4771	5308	5372
Sickle-cell/HB-C disease with crisis (282.64)	113	113	211	181
Other sickle-cell disease with crisis (282.69)	592	495	663	655
Continuous invasive mechanical ventilation for <96 consecutive hours (96.71)	162	176	192	136
Continuous invasive mechanical ventilation for ≥96 consecutive hours (96.72)	123	102	115	129
Exchange transfusion (99.01)	347	333	373	436
In-hospital mortality(total number)	88	69	142	93
In-hospital mortality as % of all hospitalizations	1.39%	1.24%	2.20%	1.42%

Note: For 47 hospitalizations, information on discharge quarter was not available.

## Discussion

Advances in medical technology, immunizations, infection control, improved patient education, and multidisciplinary care have led to an increasing number of adults living with sickle cell disease [12], [13]. Although it is known that disease severity and mortality rate are much higher in adults with sickle cell disease and acute chest syndrome compared to children [2], [6], [11], current national estimates of hospital resource utilization and outcomes in adults with acute chest syndrome are lacking. To our knowledge, our study is the largest cohort of hospitalized adult patients with sickle cell disease and acute chest syndrome whose outcomes were assessed using a multitude of patient and hospital level characteristics at a national level.

Consistent with prior studies [3], [14], we showed that adults with Hb-SS phenotype accounted for the highest rate of acute chest syndrome hospitalizations. Further, in the present study, although, Hb-SS disease with crisis accounted for the majority of hospitalizations, neither the in-hospital mortality nor the length of stay were influenced by the ***genotype*** of sickle cell disease. This could suggest that, rather than the genotype, it is the severity of acute chest syndrome and resulting respiratory failure (as evidenced by the need of mechanical ventilation) that influences mortality.

In our study, acute respiratory failure requiring mechanical ventilation was found in 4.6% of hospitalizations with an overall all cause in-hospital mortality rate of 1.6% which is lower in comparison to the 13% mechanical ventilation and 3% mortality in the National Acute Chest Syndrome Group (NACS-671 episodes) study [5], [18]. This lower mortality could be due to the advances in medical care and management strategies over the past decade. Interestingly, the mortality for patients with acute chest syndrome patients requiring mechanical ventilation in the NACS study was better than the overall mortality rate for patients with acute chest syndrome (19% compared to approximately 30%, respectively). It is very intriguing to note that, in our study, any need for mechanical ventilation was an independent predictor of mortality, with the odds of mortality being highest in the first 96 hours. Our results are consistent with a single center intensive care unit experience of higher risk of mortality in invasively mechanically ventilated patients (12–52 years) with acute chest syndrome [29]. Our results contrast those of a recent study in adolescents and young adults (16–25 years) with sickle cell disease admitted with acute chest syndrome in that although nearly 45% of those patients required mechanical ventilation, the in-hospital mortality was 0.6% of all hospitalizations [7].

The mean length of hospitalization in our study was 7.8 days which is lower compared to 10.5 days in the NACS study [18]. In our study, the predictors of increased LOS included mechanical ventilation (≥96 hours), need for exchange transfusion and increased co morbid burden, factors that are intuitively anticipated to cause increased LOS. It is of interest to note that in our study for every 1 year increase in age, the LOS was lower, although marginally low. A prior study using similar national administrative database found that older children with sickle cell disease and vaso-occlusive crisis had a longer LOS [25].

Prior studies have demonstrated that both simple and exchange transfusions alleviate organ dysfunction in patients with sickle cell crisis and acute chest syndrome [5], [15], [16], [17]. Although, no definitive randomized controlled trials exist to evaluate the timing of blood transfusion (simple or exchange) in acute chest syndrome in adults, most experts recommend no transfusions for mild, simple transfusions for moderate and exchange transfusions for severe acute chest syndrome. In our study, 6% of the hospitalizations received exchange transfusion and after adjustment of known confounding factors, exchange transfusion was not an independent predictor of in-hospital mortality, although it did lead to a 40% increase in length of stay. This is in sharp contrast to the exchange transfusion rate of 3% in adolescents/young adults hospitalized for acute chest syndrome [7]. The timing and impact of exchange transfusion on outcomes in the adult population merits further research.

A prior study showed that hospitals with low volume of admissions for sickle cell disease had higher mortality rates compared to high volume hospitals [24], a finding that is true across other disease processes as well [26], [27]. In contrast, our study found that a multitude of patient and hospital level factors such as increasing age, race, gender, co-morbid conditions, insurance, teaching status, hospital bed size or region did not influence the in-hospital mortality rate, further underscoring that the severity of illness and need for mechanical ventilation are more pertinent factors. No prior study has looked at seasonal variations in outcomes for sickle cell disease patients with acute chest syndrome. In the present study, the in-hospital mortality ranged from a low of 1.24% (April–June) to a high of 2.20% (July–September). The impact of viral infections (which have seasonal variation) on sickle cell acute chest syndrome hospitalization outcomes merits further research.

The particular strength of our study rests on the size and breadth of our sample. NIS is the largest all-payer hospital discharge database in United States and represents experiences beyond a single center. The outcomes assessed in this large cohort of patients reflect the impact of advances in medical care in the given time frame. Identifying predictors of worse outcomes is useful in devising preventive management strategies to optimize outcomes. There are, however, several methodological limitations to this study. Due to its retrospective nature, the cause and effect relationship between the independent variables and occurrence of a particular event cannot be firmly established. There are also inhered limitations associated with the use of a large secondary hospital discharge administrative datasets. Nevertheless, a recent study demonstrated the effective use of a large multi-institute administrative dataset in assessing the outcomes of adolescents and young adults with acute chest syndrome [7]. In addition, prior studies have shown that the combination of administrative data with certain clinical data can be used efficiently to stratify surgical risk [19], [20]. Although we used a multivariable regression analysis to account for the confounding effects of patient and hospital level variables, the risk adjustment performed is not comprehensive owing to the lack of adequate patient level data and severity of the disease process (mild, moderate or severe acute chest syndrome) in the NIS dataset. However, a number of chronic conditions beyond sickle cell disease and admission type (elective, urgent) were used for disease severity adjustment. Also, the nature of the dataset precludes us from assessing the impact of certain factors that are known to influence outcomes such as long term treatment with hydroxyurea, which has been shown to decrease the acute chest syndrome frequency and mortality [21], [22], [23]. Hospital readmissions of adult patients with sickle cell anemia are common and are associated with higher mortality [28]. The lack of patient identifiers precludes us from identifying readmissions in our study. Finally, our study results indicate that those with continuous invasive mechanical ventilation of <96 hours duration were associated with a very high odds of in-hospital mortality. The reasons for high mortality rates in this cohort are multifold and include: severity of acute chest syndrome, time of starting mechanical ventilation, or death during mechanical ventilation. The NIS dataset does not provide information on the severity of acute chest syndrome, event leading to death, or time of starting the mechanical ventilation. Consequently, we are unable to examine these factors.

Despite these limitations, our study reveals that acute chest syndrome is not an infrequent cause of hospitalization in adults with sickle cell disease. There was a gradual yearly increase in the number of hospitalizations due to acute chest syndrome, and an increase in mechanical ventilation in such patients. Our findings lend support to the need for better understanding the factors predisposing to respiratory failure in this growing cohort of the population.

## Conclusions

In this large cohort of hospitalized adults with sickle cell disease and acute chest syndrome, the need for mechanical ventilation predicted higher mortality rates and increased hospital resource utilization. Identification of risk factors may enable optimization of outcomes.
